# Underrecognition of Dengue during 2013 Epidemic in Luanda, Angola

**DOI:** 10.3201/eid2108.150368

**Published:** 2015-08

**Authors:** Tyler M. Sharp, Rosa Moreira, Maria José Soares, Lúis Miguel da Costa, Jennifer Mann, Mark DeLorey, Elizabeth Hunsperger, Jorge L. Muñoz-Jordán, Candimar Colón, Harold S. Margolis, Adelaide de Caravalho, Kay M. Tomashek

**Affiliations:** Centers for Disease Control and Prevention, San Juan, Puerto Rico, USA (T.M. Sharp, E. Hunsperger, J.L. Muñoz-Jordán, C. Colón, H.S. Margolis, K.M. Tomashek);; Field Epidemiology and Laboratory Training Program–Centers for Disease Control and Prevention, Luanda, Angola (R. Moreira);; Ministry of Health of Angola, Luanda (M.S. Soares, L.M. da Costa, A. de Caravalho);; Center for Global Health–Centers for Disease Control and Prevention, Luanda (J. Mann);; Centers for Disease Control and Prevention, Fort Collins, Colorado, USA (M. DeLorey)

**Keywords:** Dengue, Angola, epidemic, cluster, surveillance

## Abstract

During the 2013 dengue epidemic in Luanda, Angola, 811 dengue rapid diagnostic test–positive cases were reported to the Ministry of Health. To better understand the magnitude of the epidemic and identify risk factors for dengue virus (DENV) infection, we conducted cluster surveys around households of case-patients and randomly selected households 6 weeks after the peak of the epidemic. Of 173 case cluster participants, 16 (9%) exhibited evidence of recent DENV infection. Of 247 random cluster participants, 25 (10%) had evidence of recent DENV infection. Of 13 recently infected participants who had a recent febrile illness, 7 (54%) had sought medical care, and 1 (14%) was hospitalized with symptoms consistent with severe dengue; however, none received a diagnosis of dengue. Behavior associated with protection from DENV infection included recent use of mosquito repellent or a bed net. These findings suggest that the 2013 dengue epidemic was larger than indicated by passive surveillance data.

## Abstract 2

Case detection should be improved by instituting routine laboratory-based surveillance for acute febrile illnesses in Africa.

Dengue in Angola

Dengue is a potentially fatal acute febrile illness caused by any of 4 mosquito-transmitted dengue viruses (DENV-1–4). The disease is endemic throughout the tropics (*1*), but is underrecognized in sub-Saharan Africa (*2*,*3*), where an estimated 64 million DENV infections occurred in 2010 (*4*). Although dengue was identified in travelers returning from Angola in the 1980s (*5*), locally acquired cases had not been reported until an outbreak in 2013 that was initially thought to have resulted from importation of DENV by immigrant workers from Asia. However, the only virus detected during the outbreak was a strain of DENV-1 that molecular epidemiologic analysis indicated had been circulating in western and west-central Africa for roughly 4 decades (*6*–*9*), demonstrating regional endemicity of dengue.

During the 2013 epidemic, the Angola Ministry of Health was notified of a total of 1,214 dengue case-patients, nearly all (98%) of whom resided in the capital, Luanda, which has an estimated population of 3–14 million (Angola Ministry of Health and World Health Organization, unpub. data). Serum specimens from suspected cases were tested with a dengue rapid diagnostic test (RDT; SD BIOLINE Dengue Duo, Standard Diagnostics, Haryana, India), and positive cases were defined by detection of nonstructural protein 1 antigen, anti-DENV IgM, or both. In total, specimens from 811 (67%) persons with suspected dengue tested RDT-positive, including those from 246 (30%) hospitalized patients and from 11 (1.4%) patients who died. The highest weekly incidence occurred during May 17–23, 2013, when 125 cases were reported, of which 101 (81%) were RDT-positive.

Dengue is a focal disease, and cases frequently cluster around the households of infected persons (*10*,*11*). Previous household-based cluster investigations in Indonesia (*12*), Nicaragua (*13*), Thailand (*14*), and Vietnam (*15*) demonstrated DENV infection rates of 2.2%–12.4% among persons residing within 10–100 m of index case-patients. These studies enabled detection of unrecognized dengue cases and identification of household risk factors for DENV infection, such as the presence of uncovered water storage containers (*12*) and lack of piped household water supply (*14*). Household-based cluster investigations are therefore a useful tool to estimate the extent of dengue in regions where case reporting may be suboptimal and can also facilitate identification of local risk factors for DENV infection.

## Methods

We conducted household-based cluster investigations in Luanda to detect unreported cases and identify demographic characteristics and household and behavioral risk factors for infection. Clusters consisted of households located within a 25-m radius of the following: 1) residences of dengue case-patients who sought medical care, were reported as having a suspected dengue case, and tested positive by RDT (case clusters); or 2) randomly selected households from throughout Luanda in which no known dengue case-patient resided (random clusters).

Case clusters were identified by contacting RDT-positive dengue case-patients or their parents and querying whether they were available for a household visit, which was made within 30 days of the index case-patient’s reported date of illness onset. Case clusters were studied even if the index case-patient did not participate in the investigation. 

The protocol for selection of random clusters was as follows: 1) randomly selecting and traveling to 1 of 8 regions of Luanda; 2) spinning a 1-sided object (e.g., pen, bottle); 3) traveling in the indicated direction for ≈30 min by automobile without accounting for the degree of traffic congestion; 4) parking the automobile and spinning the 1-sided object again; 5) traveling by foot in the indicated direction for ≈5 min; 6) again spinning the 1-sided object; and 7) offering participation in the investigation to the nearest household in the direction indicated by the object. If the selected household was unoccupied or declined participation, the team returned to the automobile and repeated the process.

The head of the household in case and random clusters was informed of the purpose of the investigation. Households were not revisited if the head of household was unavailable. If heads of household agreed to participate in the investigation, all available household members were offered the opportunity to participate, which included the following: 1) completing a questionnaire that collected information on demographics, medical history, and mosquito avoidance strategies; and 2) providing a serum specimen for dengue diagnostic testing. Heads of household completed an additional questionnaire regarding household characteristics. All communications and questionnaires were in Portuguese. The cluster study was conducted during June 28–July 2, 2013. Specimens were processed on the day of collection, stored at −20°C, and shipped on dry ice to the Centers for Disease Control and Prevention (CDC) Dengue Branch (San Juan, Puerto Rico), for dengue diagnostic testing by real-time reverse transcription PCR (rRT-PCR) (*16*) and anti-DENV IgM capture ELISA (InBios International, Inc., Seattle, WA, USA). Data were compiled in a Microsoft Access (Microsoft Corp., Redmond, WA, USA) database.

Participants were household members who completed a questionnaire and provided a serum specimen. Current DENV infection was defined as detection of DENV nucleic acid by rRT-PCR. Recent DENV infection was defined as detection of anti-DENV IgM by ELISA. Dengue, dengue with warning signs, and severe dengue were defined by 2009 World Health Organization guidelines (*1*).

To identify differences in characteristics between recently infected participants and uninfected participants, we fitted generalized linear models with each of the variables of interest as the predictor. Random effects were included for households nested within clusters to account for correlation. Because it was unclear whether inference could be made to the greater population of Luanda, p values were computed through a permutation test, in which DENV infection statuses were permuted within households. Thus, results are only applicable to the surveyed population.

The investigation protocol underwent institutional review at CDC and was determined to be public health response and not research. As such, institutional review board approval was not required.

## Results

Serum specimens and questionnaires were collected from 455 cluster participants (Table 1). Similar numbers of households per cluster and participants per household were included in case and random clusters. Age and sex of participants had a similar distribution in case and random clusters. No participants had evidence of current DENV infection by rRT-PCR, 41 (9%) had evidence of recent DENV infection by IgM ELISA, and 35 (8%) had equivocal IgM ELISA results and were excluded from further analysis. Age and sex distributions were similar for recently-infected case and random cluster participants. Recently-infected participants from random clusters more frequently reported having fever in the past month and less frequently reported a febrile household member in the past month.

**Table 1:** Demographic characteristics and medical history among participants in household cluster investigations of DENV infection, Luanda, Angola, 2013*

Characteristic	Random clusters, n = 26	Case clusters, n = 21
Households per cluster, median (range)	3 (1–7)	4 (1–12)
Participants per household, median (range)	3 (1–13)	4 (1–12)
All participants	247	173
Male, no. (%)	98 (42)	78 (45)
Age, y, median (range)	22 (0–94)	25 (0–79)
Participants with evidence of recent DENV infection	25 (10)	16 (9)
Male, no. (%)	11 (48)	8 (50)
Age, y, median (range)	23 (7–65)	22 (4–42)
Fever within past 30 d, no. (%)	10 (43)	3 (23)
Fever in household member within past 30 d, no. (%)	9 (47)	10 (67)

*DENV, dengue virus. N = 420; 35 participants with equivocal anti-DENV IgM ELISA results were excluded from analysis.

Of 173 participants from 67 households in 21 case clusters, 16 (9%) had evidence of recent DENV infection (Figure). Of 247 participants from 90 households in 26 random clusters, 25 (10%) had evidence of recent DENV infection. Most case (55%) and random (77%) clusters contained at least 1 recently infected participant. Approximately one fifth of case and random cluster households had at least 1 recently infected participant.

**Figure:**
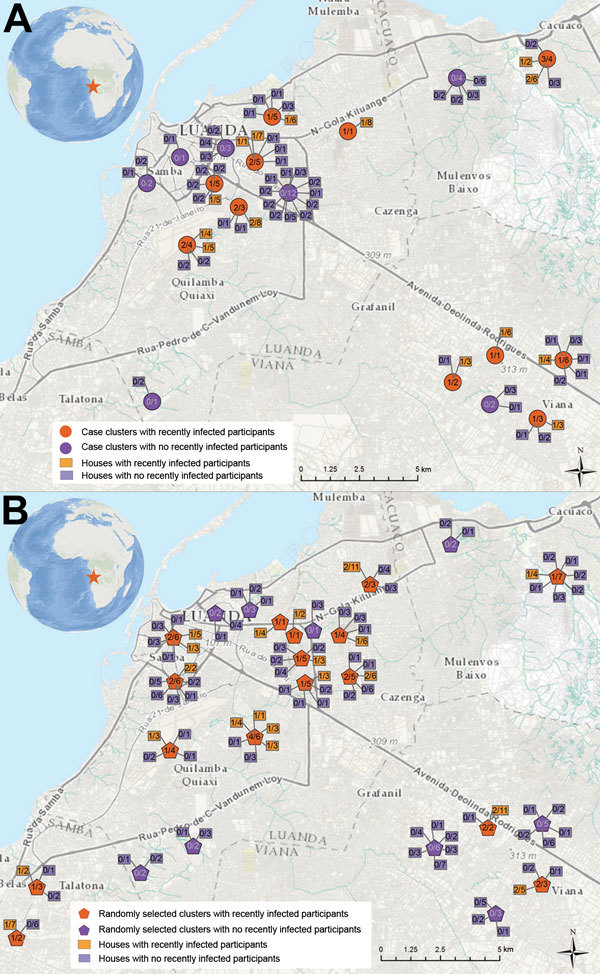
Locations of case and random cluster investigations conducted in Luanda, Angola, 2013. Clusters were conducted within a 25-m radius of A) the residences of known dengue case-patients (case clusters) or B) households in which no known dengue-case-patients resided (random clusters). The numbers in each cluster foci indicate the number of households with >1 recently infected household member per number of households included in each cluster. The numbers in each household indicate the number of recently infected participants in each household per the number of participants in each household. Coordinates were not available for 2 case clusters and 1 random cluster. Maps created by using the Geospatial Research, Analysis and Services Program (US Agency for Toxic Substances and Disease Registry, Atlanta, GA, USA).

Recently infected participants were significantly younger and had spent significantly less time in Luanda than uninfected participants (Table 2). Roughly one third of recently infected participants and also uninfected participants reported having fever in the past 30 days. Of 13 recently infected and recently febrile participants, 5 (38%) reported symptoms consistent with dengue with warning signs (severe abdominal pain) and 1 (8%) reported symptoms consistent with severe dengue (hematemesis). Seven (54%) recently infected and recently febrile participants sought medical care; 1 (14%) was hospitalized, and none reported receiving a diagnosis of dengue. Recently infected and febrile participants who sought care frequently (71%) received a diagnosis of malaria, as were uninfected, recently febrile participants who sought care (58%).

**Table 2:** Demographic, illness, behavioral, and household characteristics of participants with or without evidence of recent DENV infection that were identified though household cluster investigations, Luanda, Angola, 2013*

Characteristic	Participants with evidence of recent DENV infection, N = 41	Participants without evidence of recent DENV infection, N = 379	p value
Demographic			
Age, median (range)	14 (4–65)	24 (0–94)	**0.002**
Male sex, no (%)	19 (49)	157 (43)	0.65
Time in Luanda, y (range)	13 (3–58)	20 (0–80)	**0.01**
Medical history, no. (%)			
Fever in past 30 d	13 (33)	109 (29)	0.89
Sought medical care	7 (54)	62 (57)	0.42
Hospitalized	1 (14)	6 (10)	0.21
Diagnosis of dengue	0 (0)	4 (6)	–
Diagnosis of malaria	5 (71)	36 (58)	0.09
Diagnosis of typhoid fever	1 (14)	4 (6)	–
Other†/unknown diagnosis	1 (14)	18 (29)	–
Minor bleeding‡	2 (15)	6 (10)	0.13
Dengue with warning signs	6 (46)	23 (37)	0.57
Severe dengue	1 (8)	5 (8)	0.86
Behavioral, no. (%)			
Traveled outside of Luanda in past 30 d	0 (0)	24 (6)	0.07
Used bed net in past 30 d	2 (5)	69 (19)	**0.05**
Used repellent in past 30 d	2 (5)	46 (12)	**0.03**
Household, no. (%)			
Water supply			
Piped water supply	28 (76)	186 (67)	0.77
Public water truck	4 (11)	84 (30)	**0.04**
Other§/unknown	5 (14)	59 (21)	0.81
Had febrile household member in past 30 d	19 (56)	176 (56)	0.98
Has screened windows	1 (3)	46 (15)	0.14
Usually leave windows open	30 (79)	258 (78)	0.39
Has air conditioning	15 (39)	142 (43)	0.72
Use mosquito coils in house or yard	23 (62)	176 (53)	0.87

*Thirty-five participants with equivocal anti-DENV IgM ELISA results were excluded from analysis. DENV, dengue virus; –, insufficient numbers for permutation test. Boldface type indicates significance. †Influenza, arterial hypertension, diabetes, abortion. ‡Petechiae, epistaxis, gingival bleed. §Well, rain water.

Having used a bed net or mosquito repellent in the past 30 days were significantly associated with protection from recent DENV infection (p = 0.05 and p = 0.03, respectively; Table 2). Although most participants’ homes had piped water, delivery of household water by public water truck was also significantly associated with protection from DENV infection (p = 0.04).

## Discussion

In this investigation, ≈10% of case and random cluster participants had evidence of recent DENV infection. A possible explanation for why no participants had current DENV infection is that the cluster investigations were conducted ≈6 weeks after the apparent peak of the epidemic. Therefore, although DENV circulation may have been declining when surveys were conducted, anti-DENV IgM, which may persist for months after infection (*17*), was still detectable. This low rate of current DENV infection among cluster participants is in contrast to findings of an investigation recently conducted near the peak of a dengue outbreak in Mombasa, Kenya, in which nearly 7% of participants had evidence of current DENV infection and another 7% had recent infection (*18*).

Persons with evidence of recent DENV infection were most frequently 10–19 years of age, and this finding likely led to confounding in the observation that persons with recent DENV infection spent less time in Luanda than uninfected persons. Unfortunately, our sampling method did not allow for statistically valid age-matched comparisons. In addition, the expected natural history of DENV transmission in a disease-endemic area would support the idea that persons 10–19 years of age were more likely to have higher rates of infection than adults who had lived in Luanda for many years, had been previously infected with DENV-1, and were thus protected from infection in 2013. Taken together with molecular evidence of dengue endemicity in Angola (*7*,*8*), these observations indicate a level of dengue endemicity equivalent to that observed in the Americas (*4*), where adolescents are routinely one of the most affected age groups (*19*–*21*).

Although multiple pieces of evidence indicate that dengue is endemic in Luanda, none of the recently infected persons who sought medical care had received a diagnosis of dengue. Instead, most received a diagnosis of malaria, including 1 person who was hospitalized with an illness consistent with severe dengue. Thus, as has been observed in other regions of sub-Saharan Africa (*2*,*18*,*22*–*24*), dengue and other acute febrile illnesses may be frequently overlooked in Luanda, where malaria is in fact rare (*25*). Observations from this investigation therefore suggest that the number of RDT-positive dengue cases reported to the Ministry of Health was likely a large underestimation of the true magnitude of the 2013 epidemic.

An unexpected finding of this investigation was that the rate of detection of recently infected participants was equivalent in both case and random clusters. This finding is in contrast to those of most prior household cluster surveys, in which few persons or none were found to be recently infected in randomly selected clusters (*13*,*14*,*26*,*27*). One possible explanation for these differences is that the Luanda investigation was conducted in an urban environment soon after the peak of a large epidemic, whereas previous studies were conducted during periods of nonepidemic levels of transmission. Similar to results of this investigation, a recent 3-year cluster study conducted in urban Vietnam found that the incidence of having detectable anti-DENV IgM was twice as high in participants from case clusters than from control clusters (*15*). Alternatively, because some random clusters were surveyed on the same day and by the same teams investigating case clusters, teams may not have traveled sufficiently far from case clusters to obtain independent findings. Because dengue is a focal illness that travels in “waves” outward from urban environments (*11*,*28*), the timing and distance required to be independent from a site of known or suspected DENV transmission are unclear.

Behavior associated with protection from DENV infection included having recently used mosquito avoidance strategies (such as applying mosquito repellent or sleeping under a bed net) and delivery of household water supply by public water truck. Although the latter finding should be further investigated to both validate and explore the reasons behind its association with protection from DENV infection, use of mosquito repellent is a well-documented approach to mosquito avoidance that has been repeatedly associated with protection from DENV infection (*1*). Bed net use was previously associated with protection from DENV infection among soldiers in Somalia (*29*) but is not typically thought to be associated with protection from DENV infection because *Aedes aegypti* mosquitoes, which were the dominant vector detected during the Luanda epidemic (*8*), are most active at dusk and dawn (*1*). Bed net use may therefore be associated with protection from early morning biting. In line with this observation, a serosurvey in Mombasa, Kenya, conducted in 2013, found that leaving windows open at night was associated with DENV infection (*18*), possibly because *Aedes* mosquitoes enter the home in the evening and feed on the host in the early morning (*30*,*31*).

Strengths of this investigation include using community-level surveys to demonstrate the extent of dengue in a region where clinical awareness and reporting infrastructure were suboptimal. Moreover, the recent availability of an RDT enabled early detection of dengue cases, without which the epidemic may not have been recognized. Also, by using well-validated diagnostic tests to detect evidence of current or recent DENV infection in cluster participants, the likelihood that additional infections were missed is minimal. Conversely, infection with flaviviruses can result in cross-reactive antibody (*32*), creating the possibility of false-positive anti-DENV IgM diagnostic test results. Although the IgM ELISA used in this assay has been previously demonstrated to be highly specific for anti-DENV IgM (InBios DENV Detect IgM Capture ELISA, product insert; CDC Dengue Branch, unpub. data), it is nonetheless possible that some proportion of participants with recent DENV infection were misclassified due to false-positive diagnostic test results. 

The circulation of DENV-4 was recently detected in Luanda (*33*,*34*) and may be associated with future epidemics in the region. Because early identification and proper management of dengue patients can reduce case-fatality rates among hospitalized patients from ≈10% to <0.5% (*35*), clinical awareness of dengue should be improved in Luanda and throughout sub-Saharan Africa through clinical dengue patient management trainings (e.g., http://www.cdc.gov/dengue/training/cme.html). Case reporting should also be improved by instituting routine laboratory-based surveillance for acute febrile illnesses in Africa, which will assist in better defining the epidemiology of dengue and other emerging infectious diseases (*33*,^36^).
